# The Secretory Response of Rat Peritoneal Mast Cells on Exposure to Mineral Fibers

**DOI:** 10.3390/ijerph15010104

**Published:** 2018-01-10

**Authors:** Violetta Borelli, Elisa Trevisan, Vita Francesca, Giuliano Zabucchi

**Affiliations:** Department of Life Science University of Trieste, University of Trieste, 34127 Trieste, Italy; elisatr@inwind.it (E.T.); vita@units.it (V.F.); zabucchi@units.it (G.Z.)

**Keywords:** mast cells, asbestos, crocidolite, titanium oxide nanowires, secretory process, endocytosis

## Abstract

Background: Exposure to mineral fibers is of substantial relevance to human health. A key event in exposure is the interaction with inflammatory cells and the subsequent generation of pro-inflammatory factors. Mast cells (MCs) have been shown to interact with titanium oxide (TiO_2_) and asbestos fibers. In this study, we compared the response of rat peritoneal MCs challenged with the asbestos crocidolite and nanowires of TiO_2_ to that induced by wollastonite employed as a control fiber. Methods: Rat peritoneal MCs (RPMCs), isolated from peritoneal lavage, were incubated in the presence of mineral fibers. The quantities of secreted enzymes were evaluated together with the activity of fiber-associated enzymes. The ultrastructural morphology of fiber-interacting RPMCs was analyzed with electron microscopy. Results: Asbestos and TiO_2_ stimulate MC secretion. Secreted enzymes bind to fibers and exhibit higher activity. TiO_2_ and wollastonite bind and improve enzyme activity, but to a lesser degree than crocidolite. Conclusions: (1) Mineral fibers are able to stimulate the mast cell secretory process by both active (during membrane interaction) and/or passive (during membrane penetration) interaction; (2) fibers can be found to be associated with secreted enzymes—this process appears to create long-lasting pro-inflammatory environments and may represent the active contribution of MCs in maintaining the inflammatory process; (3) MCs and their enzymes should be considered as a therapeutic target in the pathogenesis of asbestos-induced lung inflammation; and (4) MCs can contribute to the inflammatory effect associated with selected engineered nanomaterials, such as TiO_2_ nanoparticles.

## 1. Introduction

Inhaled durable particles and fibers have been of concern to human health for over a century. Many air pollutants exert their principal effect by causing oxidative stress in cells and tissues that they come in contact with. A key event in these processes is the interaction of nanoparticles with inflammatory cells and the subsequent production of pro-inflammatory factors.

Many reports have been published on the “in vitro” interaction of different types of polluting nanoparticles/fibers with inflammatory cells and on the “in vivo” outcome of the exposure of experimental animals to different types of particulate matter. Exposure to asbestos fibers and to titanium oxide (TiO_2_) nanoparticles (NPs) is of significant relevance for human health.

Asbestos has been banned, but its effects (aggressive lung tumors and chronic lung inflammation/fibrosis) in exposed subjects is expected to reach its peak in 2025 [1]. Conversely, TiO_2_ nanoparticles (NPs) have received a much attention due to their widespread and increasing use in products such as sunscreens, cosmetics, toothpaste, pharmaceuticals, paints, plastics, self-cleaning devices, food additives, papers, solar cells, and a corrosion-protective coating in bone implants, leading to high human and environmental exposure. Generally, TiO_2_ is considered a low toxicity NP, although if it reaches the pulmonary interstitium it can exert a certain degree of cytotoxicity [2]. However, recent studies have reported that the degree of toxicity of TiO_2_ can depend on physicochemical parameters, such as the morphology of TiO_2_NPs, size, aggregation, crystal phase, and surface modifications [3,4,5]. They have been shown to be cytotoxic for both epithelial and inflammatory cells [3,6,7,8] and induce the production of reactive oxygen species (ROS) from phagocytic cells [5,6,8,9,10]. A cytotoxic effect of TiO_2_NPs for these cells (and fibroblasts) was also shown in other studies [11,12,13,14]. The pro-inflammatory power of TiO_2_NPs has also been tested in “in vivo” studies [15]. Very recently, it was also shown that TiO_2_NPs can induce an inflammatory reaction in exposed subjects who prepare TiO_2_ from ilmenite ore [16,17]. TiO_2_NPs can promote the expression and release of both T helper lymphocyte type 2 (Th2) and type 1 (Th1) cytokines [3,4,12,18,19,20]. Supporting the Th2-promoting activity of TiO_2_, it was shown that these nanoparticles can potentiate allergic reactions [21,22,23] and that intratracheally instilled TiO_2_NPs induce an increased number of mast cells (MCs) and increase interleukin 13 (IL13) secretion in the bronchoalveolar lavage (BAL) of treated rats [24]. Only in two studies have conflicting results been obtained [25,26]. It is noteworthy that these experimental models have examined different administration routes, doses employed, time of exposure, and types of TiO_2_NPs [20,27]. Apart from quantitative effects, the qualitative effects of TiO_2_ particles of different morphology and structure are very similar in terms of pro-inflammatory (i.e., ROS production, cytokine production, ingestion path) and cytotoxic activity [3,4,8,10,13], even independently of the administration route [4,12,13,20,27]. Interestingly, TiO_2_NPs and nanowires appear to be taken up by inflammatory and epithelial cells through two pathways: endocytosis and penetration, with the latter being suggested by the presence of free cytosolic TiO_2_NPs in the absence of a limiting endosome membrane [4,6,11,14,28,29]. Intriguingly, it has been suggested that asbestos fibers may also reach the cell interior by the same pathways [30,31,32].

Asbestos fibers have been widely recognized as strong pro-inflammatory and mutagenic compounds [33,34,35], although their mechanism of action is still unclear. The interaction between asbestos fibers and professional phagocytes has been widely studied in recent years [30,36,37,38,39,40,41,42,43,44,45,46,47]. When exposed to asbestos, these cells produce free radicals [34,48] and cytokines [35], ingest the fibers, undergo endoplasmic reticulum stress, and trigger the apoptotic program [49,50].

In addition to proinflammatory and cytotoxic activity, and cell penetration pathways, TiO_2_NPs and asbestos fibers also share other features. Yazdi et al. [51] have shown that TiO_2_NPs activate the NLR pyrin domain-containing 3 (NLRP3) inflammasome, which is involved in inflammation and other immune responses, leading to interleukin (IL)-1β release, and induces the regulated release of IL-1α. The same activity is also exerted by asbestos fibers [52]. As with TiO_2_NPs, asbestos fibers can provoke marked pulmonary eosinophilia, suggesting activation of Th2 immune reaction [53,54], and increase the incidence of respiratory symptoms and asthma in human beings [55]. Moreover, it was shown that amphibole fibers (including crocidolite) induce the expression of Th2-and Th1 cytokines in mouse lungs [4,11,22,23,54].

MCs have recently been identified among inflammatory cells with properties peculiar to professional phagocytes [56,57,58,59]. MCs are well known for their role in the initiation of allergic diseases [60,61] and in pathogen recognition [61,62,63,64]. Despite the fact that MCs are present, together with professional phagocytes, in the entry site of the inhaled fibers [65,66,67,68], little is known about the mast cell–NP/fiber interaction. Seminal papers have suggested that MCs are associated with interstitial fibrosis and granuloma formation induced by asbestos fibers in rats [68,69,70]. Fibrosis, which is a key event in asbestos-related disease, together with the inflammatory reaction, can be promoted by MC granule content such as tryptase, a key enzyme for profibrotic activity, and chymase, which can also act as a proinflammatory and profibrotic enzyme [71]. Mast cell chymase promotes hypertrophic scar fibroblast proliferation and collagen synthesis by activating the TGF-β1/Smad signaling pathway. These aspects potentially link MC secretory activity with premalignant stages in asbestos-related diseases.

Recently, it has been shown that MCs appear to contribute to the inflammatory and toxic effects associated with selected engineered nanomaterials [72] with fibrous structures similar to asbestos, exposure to which may lead to asbestos-like diseases in animal models [73,74]; silver nanoparticles are also capable of stimulating MC degranulation [75]. Finally, TiO_2_NPs have also been shown to stimulate a mast cell-like cell line to secrete histamine [76] and to increase the number of these cells in the trachea of treated rats [24].

Considering that MCs have been shown to be a target of TiO_2_NPs and asbestos fibers, we hypothesized that both can stimulate mature MC secretion.

In this paper, we compare the response of rat peritoneal mature mast cells (RPMCs) challenged with identical concentrations of the following mineral fibers: TiO_2_ nanowires (TiO_2_NWs), asbestos in the form of amphibole-type crocidolite (CRO) fibers, and wollastonite (WOLLA), a fibrous calcium silicate which is thought to have lower toxicity levels and low pro-inflammatory power compared to asbestos [77]. Our findings show that CRO and TiO_2_NWs are capable of triggering a dramatic secretory response in RPMCs, with the former inducing the highest activity.

## 2. Materials and Methods

### 2.1. Reagents

Triton X-100, bovine serum albumin (BSA), human chymase, human tryptase, *o*-phthalaldehyde, histamine, 3,3′,5,5′-Tetramethylbenzidine (TMB), Percoll and compound 48/80 were obtained from Sigma-Aldrich S.R.L. Milan (Italy). High-purity trypan blue (TB) (color index 23850) was obtained from Merck KGaA (Darmstadt, Germany). All other chemicals were of reagent grade.

### 2.2. Mineral Fibers

TiO_2_ nanowires were obtained from Sigma-Aldrich S.R.L. (product number: 774510). Wollastonite, as a non-asbestos silicate powder, was used as a control particulate and was a kind gift of Bal-Co. SpA (Sassuolo, MO, Italy). Wollastonite characterization was also reported by Governa et al. 1998 [77]. An Analytical Standard UICC sample of crocidolite was obtained from SPI-CHEM West Chester, PA, USA, re-suspended in phosphate buffered saline (PBS) at a final concentration of 10 mg/mL, and stored at 4 °C until use. The fiber size parameters of the asbestos UICC standard have been described in detail by Kohyama et al. [78]. The reference batch of the standard sample was Crocidolite South African 12001-28-402704-AB. All fiber types in PBS were left to sediment for 2 min to avoid larger fiber aggregates.

### 2.3. Characterization of Fibers by SEM—Energy Dispersive X-ray Spectrometry (EDX) Analysis

The fibers, resuspended in distilled water, were directly mounted onto stubs using double-sided adhesive, sputter coated with gold in a Edwards S150A apparatus (Edwards High Vacuum, Crawley, West Sussex, UK), and examined with a Leica Stereoscan 430i scanning electron microscope (Leica Cambridge Ltd., Cambridge, UK), equipped with a SEM-EDX (Oxford Instruments, Oxford, UK), and a PENTAFET PLUS TM Si(Li) detector that allowed chemical characterization. Supplementary Figures S1–S3 show the ultrastructural morphology and chemical composition of the mineral fibers employed. The chemical compositions of crocidolite and TiO_2_NWs were found to be compatible with those already reported by other authors [79,80]. As expected, WOLLA (a calcium inosilicate) was revealed to be mainly composed of calcium and silicon. The detailed morphological analysis carried out on at least 100 fibers showed that crocidolite fibers had an average length of 9.2 ± 1.5 μm (Standard Error, SE) (range minimum–maximum 2.1–35.7); wollastonite 4.2 ± 0.79 μm (SE) (range min–max 1.7–27.6); and TiO_2_NWs 2.9 ± 0.5 μm (SE) (range min–max 1.0–20.2).

### 2.4. Animals

All experimental procedures involving the use of rats were reviewed and approved by the Trieste University Animal Care and Use Committee (Protocol # 387Bor11 14 April 2011, as received from Italian Ministero della Salute on 26 April 2011) and were performed in accordance with the Italian Laws (D.L.vo 116/92 and following additions), which enforce Directive EU 86/609. All animals were housed under pathogen-free conditions with light–dark cycles, fed standard animal chow, and given free access to autoclaved distilled water from bottles.

### 2.5. Rat Peritoneal Cell Preparation, Mast Cell Purification, and Cell Viability

Wistar male or female rats (200 to 400 g) purchased from the animal facility of the University of Trieste were used. Rats were killed using CO_2_ inhalation. As judged by optical analysis of cytospin specimens (Cytospin 2, Shandon Inc., Pittsburgh, PA, USA) stained with the Diff-Quik system (Medion Diagnostics, Gmbh, Düdingen, Switzerland), highly purified (>95–97%) peritoneal mast cells (RPMCs) and contaminating peritoneal macrophages (2–5%) were obtained by centrifugation on Percoll gradient, as previously described [81]. RPMC viability (Trypan blue exclusion test) was over 95% at the beginning of the experiment. RPMCs were washed once in PBS containing 0.5% BSA, counted electronically (Coulter Counter ZBI, Luton, UK), and suspended in a buffered saline solution containing BSA (BSSA: 142 mM NaCl, 2.7 mM KCl, 0.5 mM CaCl_2_, 8 mM Na_2_HPO_4_, 10 mM KH_2_PO_4_,1.2 mM MgCl_2_, and 0.1% BSA) as previously described [81].

### 2.6. RPMC Lysate Preparation and Treatment

RPMCs (3 × 10^6^ cells/mL in BSSA) were sonicated for 2 min at maximum power in a Bandelin Sonopuls Sonifier UW2070 (Berlin, Germany) which ensures the near complete disruption of both cells and organelles, and subsequently incubated for 30 min at 37 °C with mineral fibers (100 μg/mL). The quality of the lysate was monitored by checking that, after centrifugation (15 min at 250× *g*) of the untreated lysate, less than 17% of the enzyme activity was found in the pellet fraction. Following incubation with fibers, enzymatic activities for chymase (CHY), tryptase (TRY) and β-hexosaminidase (β-hexo) were measured (without detergent which could in theory interfere with fiber absorption capacity) in lysates, to evaluate a possible inhibitory or stimulatory effect induced by fibers. Subsequently, the mixture was centrifuged 15 min at 250× *g* at 4 °C and the supernatant and the pellet (containing all the fibers added) were carefully collected. The enzyme activities were measured again in these fractions to assess their possible fiber-association.

### 2.7. RPMC Fiber Interaction

RPMCs (3 × 10^6^ cells/mL in BSSA) were incubated (5–30 min, as indicated, at 37 °C) alone, with mineral fibers (100 μg/mL), or stimulated to degranulate by adding compound 48/80 to a final concentration of 10 µg/mL. To stop the interaction, tubes were rapidly chilled in ice. Cytospin specimens of the incubation mixtures were stained with the Diff-Quik system to assess morphologically the degranulation process. Cells were then pelleted by centrifuging 10 min at 200× *g* at 4 °C, the supernatant (SN) was carefully collected, and the cell pellet (P) was resuspended in the same volume of BSSA.

### 2.8. Release of Granule Components

The amount of released granular mediators was determined by measuring the activities of β-hexo, TRY, CHY, and the presence of histamine in supernatants (SN) and pellets (P). The enzymatic activities in the cell fractions (solubilized with 0.05% Triton X-100) were determined using the following substrates: 4-nitrophenyl *N*-acetyl-β-d-glucosaminide for β-hexo, *N*-(*p*-Tosyl)-Gly-Pro-Lys 4-nitroanilide (0.25 mM final concentration) for TRY, and *N*-succinyl-Ala-Ala-Pro-Phe-*p*-nitroanilide (0.2 mM final concentration) for CHY. The supernatant and pellet enzymatic activities were assayed in triplicate in 96-wells plates, and measured in an ELISA reader (Multiskan™ FC Microplate Photometer; Thermo Fisher Scientific, Waltham, MA, USA) at 405 nm. Histamine content was assessed by a fluorometric assay in a Perkin Elmer spectrophotofluorimeter (λ5) using *o*-phthaldehyde (OPT) reaction as previously described [82]. The extent of secretion was calculated by considering the total enzyme activity or histamine amount (SN + P) as 100%.

### 2.9. MC Granule Preparation and Granule–Fiber Interaction

Intact mast cell granules enveloped by their individual perigranular membranes were obtained by mild sonication (1 min at 50% power in Bandelin Sonopuls Sonifier UW2070, Berlin, Germany) of the MC suspension as described by Lindstedt and Kovanen [83]. Granule remnants were obtained by weak 48/80 stimulation (15 min with 1 μg/mL 48/80) of MC as previously described [83]. Granule structures were incubated with 100 μg/mL mineral fibers for 30 min at 37 °C, and then processed for scanning electron microscopy.

### 2.10. Optical and Ultrastructural Scanning Electron Microscope Analysis (SEM)

At the end of the incubation, cell/fibers or granule/fibers suspensions were stained with the Diff Quik System or fixed in 2% glutaraldehyde in 0.1 sodium cacodylate buffer (pH 7.4) for 30 min. Fixed cells were washed twice in sodium cacodylate buffer (0.1 M pH 7.4) placed on glass coverslips (previously coated with poly-l-lysine) for 1 h, and then processed for scanning electron microscopy (SEM). The procedure to analyze the samples by SEM has been previously described elsewhere [31]. Samples rinsed in PBS were dehydrated in ascending ethanol concentrations and transferred in 100% ethanol to a critical point dryer (Bal-Tec; EM Technology and Application, Furstentum, Liechtenstein) and dried through CO_2_. Coverslips were mounted on aluminum sample stubs and gold-coated by sputtering (Edwards S150A apparatus; Edwards High Vacuum, Crawley, West Sussex, UK). SEM images were obtained using a Leica Stereoscan 430i scanning electron microscope (Leica Cambridge Ltd., Cambridge, UK).

### 2.11. Transmission Electron Microscope Analysis (TEM) Fibers

RPMCs (3 × 10^6^ cells in BSSA) were challenged with mineral fibers (100 µg/mL) for 30 min at 37 °C, and were subsequently fixed for 30 min at room temperature in a solution of 1.5% glutaraldehyde (Serva, Heidelberg, Germany) in 0.1 M sodium cacodylate buffer (pH 7.4) containing 0.03 M CaCl_2_. As previously described [58], samples were washed twice with sodium cacodylate buffer (pH 7.4) and then post-fixed with 1% OsO_4_ for 1 h at 4 °C. Post-fixed cells were dehydrated with a graded ethanol series ending with 100% ethanol and then embedded in Dow epoxy resin (DER332; Unione Chimica Europea, Milan, Italy) and DER732 (Serva, Heidelberg, Germany). Ultrathin sections were prepared with an Ultrathome III (Pharmacia-LKB, Uppsala, Sweden) and double stained with uranyl acetate and lead citrate. Serial sections were obtained by spanning 120–720 nm from the initial level. All the sections were analyzed using a Philips EM208 transmission electron microscope (Philips, Eindhoven, The Netherlands) equipped with a Quemesa Camera (Olympus Soft Imaging Solutions, Munster, Germany).

### 2.12. Peroxidases

Myeloperoxidase (MPO) and eosinophil peroxidase (EPO) were obtained from human blood granulocytes as described previously [84]. Purified MPO had a ratio of absorption in the Soret region 428/280 nm (ratio of Reinheitzahl (Rz)) commonly used as a criterion of purity for heme peroxidases) of 0.8, which indicates a high level of purity; its concentration in 25 mM phosphate buffer (pH 7.0) was 1.6 mg/mL (10.7 µM). Purified EPO (protein concentration, 0.32 mg/mL [5 µM] in 25 mM phosphate buffer (pH 7.0) had the typical absorbance spectrum reported previously and had an Rz of 1.10. The enzymatic activity of MPO and EPO was analyzed using the TMB method [85].

### 2.13. Isoelectric Point (IP)

The theoretical IPs for the enzymes considered in this paper are those reported in the UniProt universal protein knowledgebase web site (Nucleic Acids Res. 45: D158–D169 (2017)).

### 2.14. Statistical Analysis

Statistical significance was tested by a two-tailed Student’s *t* test for paired samples using GraphPad Prism 5.0 (GraphPad Software, La Jolla, CA, USA). When assessing the statistical significance of increased enzyme activity, one sample *t* test was used to calculate to what extent the mean values differed from a hypothetical value of 100. Values of *p* ≤ 0.05 were considered statistically significant.

## 3. Results

### 3.1. Morphological Analysis of Mineral Fiber–RPMC Interaction by Light and Electron Microscopy

**Figure 1:**
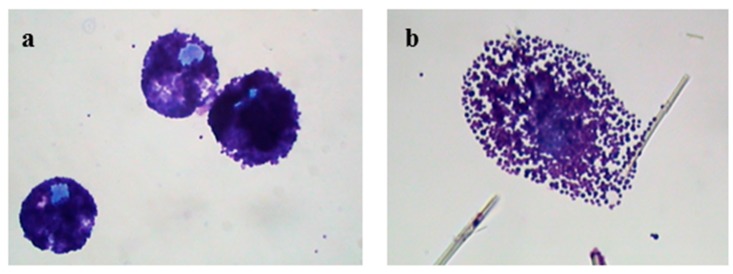

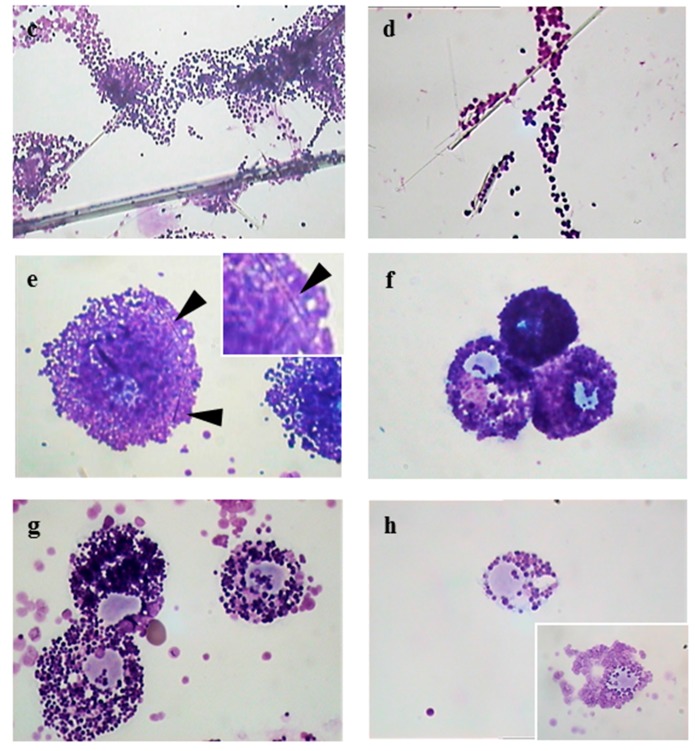
Light microscope appearance of rat peritoneal MCs (RPMCs) exposed to mineral fibers or stimulated with compound 48/80. (**a**) Unstimulated RPMCs after 30 min of incubation; (**b**,**c**) RPMCs exposed to 100 µg/mL of crocidolite fibers after 5 or 30 min of incubation; (**d**) isolated RPMC membrane-covered granules incubated with 100 µg/mL of crocidolite for 30 min; (**e**) an RPMC exposed to 100 µg/mL TiO_2_ nanowires (NWs) after 30 min of incubation, arrowheads show intracellular nanowires and in the magnified inset the arrowhead shows a nanowire inside the cell; (**f**) RPMCs exposed to 100 µg/mL of wollastonite fibers after 30 min of incubation. ((**g**,**h**), inset) RPMCs stimulated with compound 48/80 10 µg/mL after 5 or 30 min incubation; the pictures are representative of at least ten different experiments. Cytocentrifuge-prepared samples were stained with the Diff-Quik system. Original magnification = 1000×.

**Figure 2:**
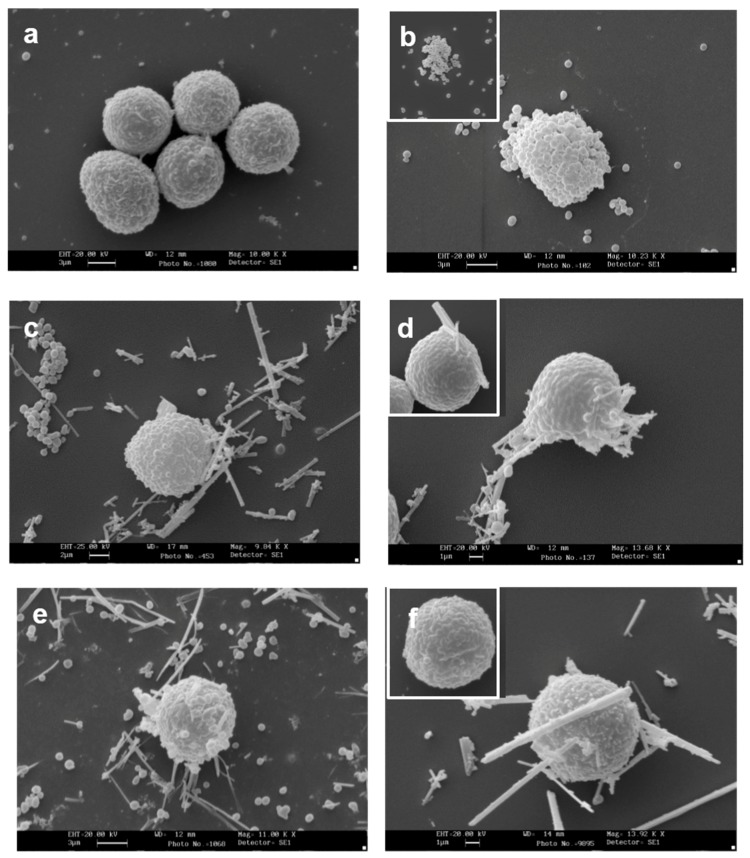
Scanning electron microscope appearance of RPMCs exposed to mineral fibers or stimulated with compound 48/80. (**a**) Unstimulated RPMCs after 30 min of incubation; (**b**) RPMCs exposed to 10 µg/mL compound 48/80 for 30 min: a large number of granules are secreted and the cell surface shows many protruding granules, while the inset shows an RPMC ghost induced by 48/80; (**c**,**d**) RPMC exposed to 100 µg/mL crocidolite for 30 min: an evident secretory response is shown, fiber-bound free granules as well as granule clusters can be seen, and numerous granule profiles protrude from the cell surface. ((**d**) and inset) Asbestos fibers can be seen, entrapped by the RPMC; the fibers may have reached the cell interior by either penetration or frustrated phagocytosis; (**e**) an RPMC exposed to 100 µg/mL TiO_2_NWs for 30 min. Note that numerous fibers interact with cell and free granules; ((**f**) and inset) an RPMC exposed to 100 µg/mL wollastonite fibers for 30 min. Note that despite many fibers being deposited on the RPMC surface there is no sign of degranulation. Magnification: bars in (**a**,**b**,**e**) = 3 µm; bars in (**c**) = 2 µm; in (**d**,**f**) = 1 µm.

Figure 1a shows the appearance of a population of unstimulated rat mast cells. They appear compact and maintain this morphology up to 30 min of incubation. After as little as 5 min of exposure, crocidolite (CRO) fibers induce RPMC degranulation. The interacting cells become enlarged and show scattered granules, which appear to be independent of one another and are projected to the cell periphery (Figure 1b). The process reaches its maximum after 30 min of incubation, when most of the RPMCs appear to be disrupted (Figure 1c). As shown in Figure 1b,c, secreted granules can be seen adhering to the fibers. The affinity of this binding was shown by isolating RPMC membrane-covered granules and incubating them directly with CRO. Figure 1d shows isolated granules adhering to asbestos fibers. CRO seems to trigger an RPMC explosion, with expulsion of many granules/granule remnants at the same time. Most fibers displayed large numbers of bound granules. Figure 1e shows the appearance of an RPMC incubated for 30 min with TiO_2_NWs: two nanowires (Figure 1e inset) are visible inside the RPMC. Even in this case the degranulation process is evident: the cells exhibit enlarged and scattered granules, suggesting that TiO_2_NWs can also induce RPMC degranulation. On the contrary, incubation with WOLLA did not result in RPMC degranulation. In Figure 1f cells appear to maintain their unstimulated morphology even after the end of the incubation. Compound 48/80, a well-known inducer of MC secretion, also had a significant effect. After 5 min, it induced the progressive degranulation of the RPMCs (Figure 1g) leading, eventually, to the formation of RPMC ghosts (Figure 1h and inset). In order to gain further insight into the mechanism of mineral fibers-RPMC interaction, we carried out scanning electron microscope (SEM) and transmission electron microscope (TEM) analyses. Figure 2a shows the SEM appearance of unstimulated RPMCs, with some granule profiles protruding beneath the plasma membrane level. As expected, stimulation for 30 min with 48/80 elicited degranulation (Figure 2b and inset). After 30 min incubation, CRO stimulated RPMCs to secrete their granules, which were subsequently found to be associated with the fibers themselves (Figure 2c arrowhead). In some cases (Figure 2d and inset), single fibers were seen entrapped in the RPMCs. Figure 2e shows that RPMCs were also able to entrap TiO_2_NWs and undergo degranulation. Even in this case, the secreted granules appeared to bind to extracellular NWs (Figure 2e). Figure 2f (and inset) shows the appearance of an RPMC exposed to WOLLA for comparison. In this case, the cells maintained their unstimulated morphology during the entire incubation time, despite the large number of WOLLA fibers deposited on their surface. Free granules were only rarely seen.

In all these cases the extracellular granules could derive from either an active degranulation process or from direct rupture of the RPMC followed by the passive release of membrane-covered granules. To provide more insight into this problem, we isolated membrane-covered granules and granule remnants and challenged them firstly with the more active mineral fiber, crocidolite. The results (Supplementary Figure S4 panel a) shows the appearance of membrane-covered granules compared to panel b, which shows granule remnants isolated following secretion induced by 48/80. The granules, immediately fixed after isolation, show a very similar morphology. However, the diameter of granule remnants was slightly but highly and significantly (*p* < 0.005) increased with respect to membrane-covered granules (0.70 mm ± 0.02 SE vs. 0.80 mm ± 0.02 mean ± SE, with more than 200 observations carried out on 15 different SEM fields). The major difference was adhesion to CRO fibers. Detailed counts on at least 10 different SEM fields revealed that on average 0.24 granule remnants and 2.2 membrane-covered granules were bound to one CRO fiber. Panel C and E clearly show a difference. Since more active adhesion of granule fibers was found with membrane-covered granules, we employed the latter to further study the outcome of their incubation with mineral fibers.

**Figure 3:**
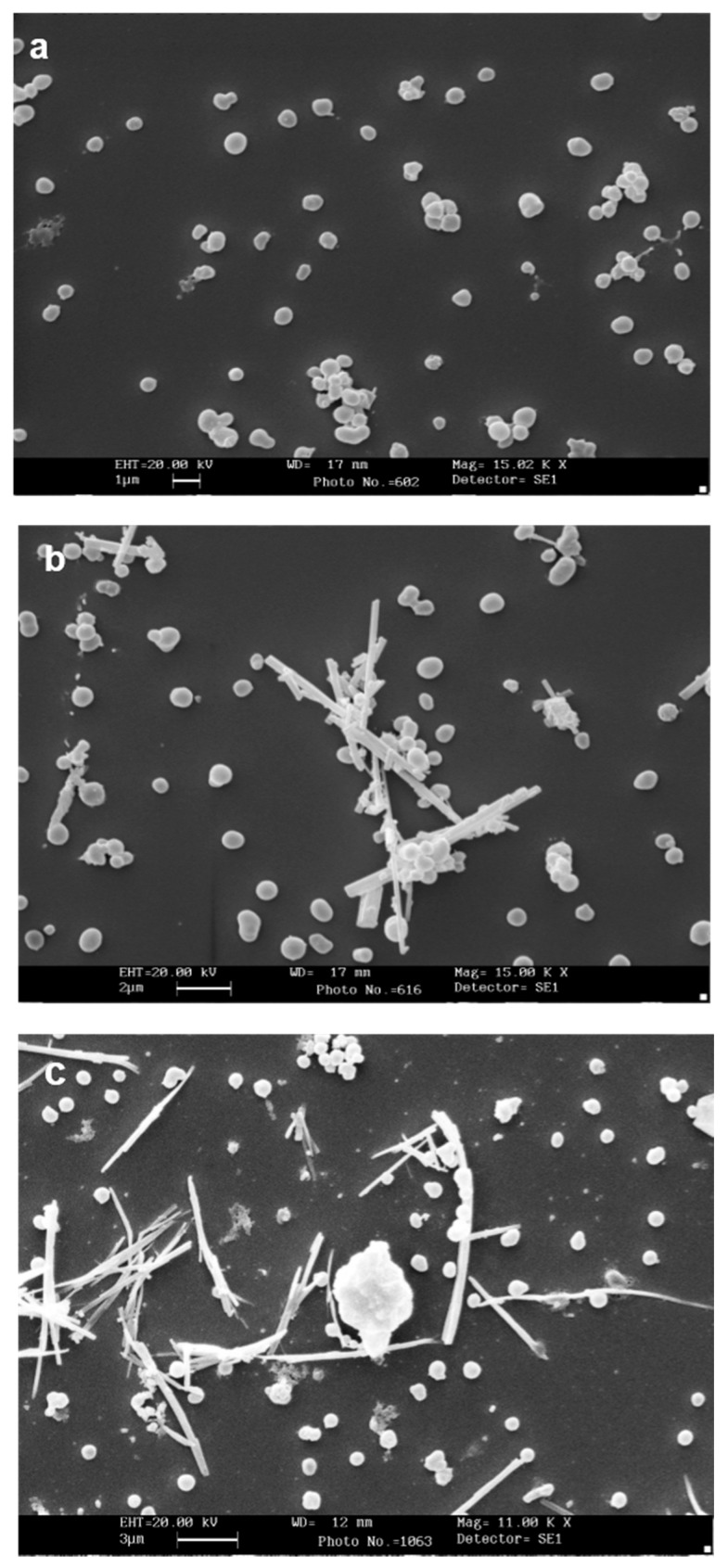
Interaction of RPMC-isolated granules with crocidolite as observed by SEM. (**a**) Isolated membrane-covered granules alone; (**b**) isolated membrane-covered granules incubated with 100 µg/mL of crocidolite fibers; (**c**) isolated membrane-covered granules incubated with 100 µg/mL of TiO_2_NWs. A fiber-bound cluster of granules can be seen. Magnification: bars in (**a**) = 1 µm; (**b**) = 2 µm; (**c**) = 3 µm.

Figure 3 shows the interaction of CRO and TiO_2_NWs with isolated MC membrane-covered granules. This interaction, in agreement with the findings obtained by light microscope analysis, appeared to be remarkably consistent (Figure 3b). Granule-free CRO was rarely observed. TiO_2_NWs displayed lower, yet still consistent, interaction with isolated granules (Figure 3c).

**Figure 4:**
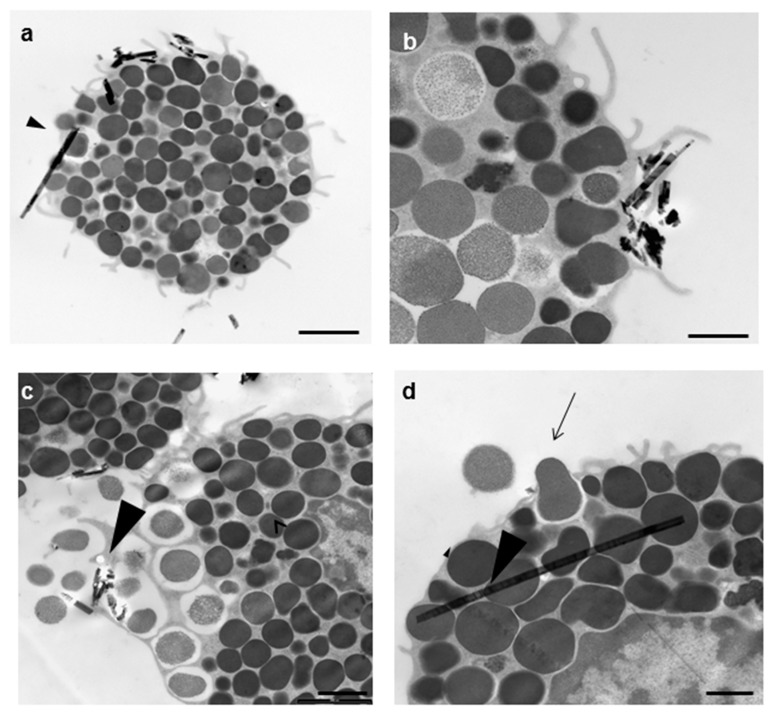
Transmission electron microscopy appearance of an RPMC exposed to 100 µg/mL of crocidolite fibers. After 30 min of incubation, the interacting fibers appear to undergo ingestion (**a**,**b**) or are free in the cytosol (arrowhead in (**a**,**d**)); (**c**) a degranulation process is evident where asbestos fibers are present; (**d**) the RPMC releasing a single granule (arrow). Magnifications: bar in (**a**) = 2 µm; bar in (**b**) = 1 µm; (**c**) = 2 µm; (**d**) = 0.5 µm.

**Figure 5:**
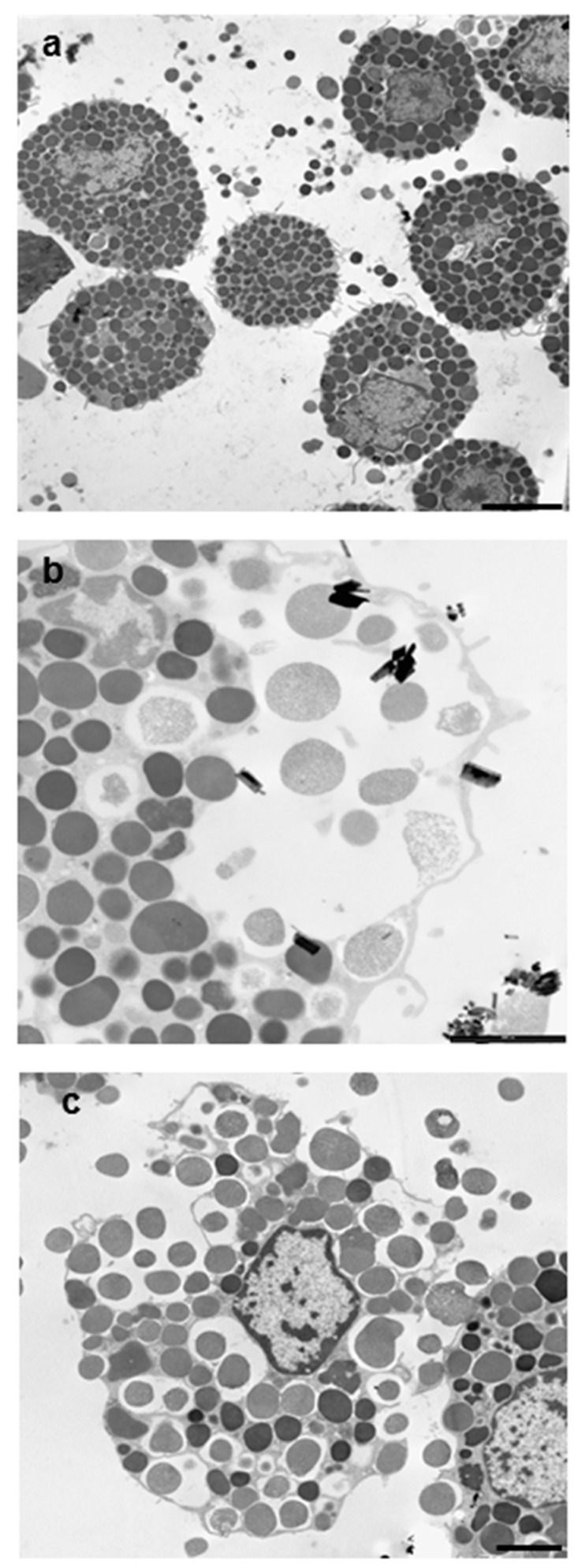
Transmission electron microscopy appearance of RPMC exposed to 100 µg/mL of crocidolite fibers. (**a**) The unstimulated RPMC population; (**b**,**c**) a crocidolite-stimulated RPMC. After 30 min of incubation the secretory process appears to follow two pathways. In some cases, the secretion appears to follow the conventional compound exocytosis pathway (**b**), which is characterized by multiple granule fusion and formation of large vacuoles, which will subsequently release the granule content in one step and give rise to granule remnants. On bottom right a granule remnant adhering to asbestos fibers is shown. In other cells the process follows cell disruption with intact granule expulsion (**c**). Magnifications: bar in (**a**,**c**) = 2 µm.

Since the more potent effect was registered with CRO fibers, these were used to stimulate RPMCs for TEM analysis. The objective was to investigate whether, as already shown for TiO_2_ nanoparticles [5,7,31], asbestos fibers can also penetrate RPMCs. CRO interacts with the RPMC surface in different ways: some fibers are embraced by MC pseudopodia and presumably ingested (Figure 4a–c), while some simply adhere, and the majority seem to be included in the RPMC cytosol (arrowhead in Figure 4a,d), while only rarely can a phagosome be seen surrounding them. The appearance of TEM suggests that the secretion can be carried out through: (1) active single granule secretion (Figure 4d arrow) and the conventional compound exocytosis pathway (Figure 5b), when the granule membranes fuse to each other, forming large vacuoles whose content is subsequently released extracellularly in one step; and (2) following membrane rupture and passive granule expulsion (Figure 5c). It is worth noting that the ultrastructure of asbestos-challenged RPMCs showed a weaker reaction to fiber exposure when analyzed by TEM and SEM than that observed by light microscopy. Most of the cells were intact and only rarely exhibited an explosive reaction. Almost all the RPMCs interacting with the fibers showed signs of activated secretion. It can be excluded that the nude asbestos fibers visible in the cytosol are artefacts introduced by the preparation and processing of the cell sections for TEM analysis, since serial sections of the same cell show the presence of the same fiber spanning either 120 or 720 nm in depth (see Supplementary Figures S5 and S6). The analysis of an eosinophil gave the same result (Supplementary Figure S7).

The morphological appearance of MCs stimulated by CRO or TiO_2_NWs suggests that a large amount of granule content has been secreted into the extracellular medium. To confirm this hypothesis, we analyzed the stimulated RPMC population for the presence of granule components in the incubation medium.

### 3.2. Quantization of Secretion of Granules Components Induced by Mineral Fibers

In order to monitor the secretory process induced in MCs by mineral fibers, we separated the supernatant of the stimulated cells from the cell pellet and measured the enzymatic activities of CHY, a serine protease that accounts for half of the protein content of RPMC secretory granules (62), TRY, β-hexosaminidase (β-hexo), and histamine, which are widely accepted markers of mast cell secretion (63).

**Figure 6:**
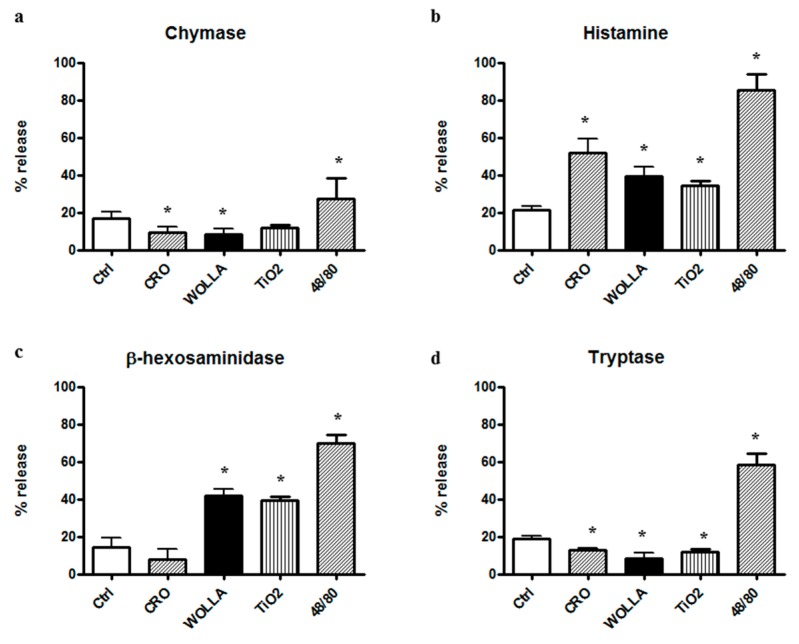
Enzyme secretion from mineral fiber (100 µg/mL) or 48/80 compound (10 µg/mL)-stimulated RPMCs. Values are the mean ± SD of at least four different experiments. The extent of secretion, i.e., the percentage of enzyme activity in the supernatant, was calculated taking total enzyme activity (supernatant + pellet) as 100% (mean value ± standard deviation (SD): 0.074 ± 0.006 for chymase, 0.079 ± 0.006 for β-hexosaminidase (β-hexo) Optical Density (OD)/30 min and 0.060 ± 0.005 OD/30 min for tryptase). In the case of histamine, the total (supernatant + pellet) fluorescence arbitrary units were considered as 100%. See text for details. In the case of β-hexosaminidase, the control value did not differ significantly from that obtained with crocidolite stimulation. In all other cases, the difference between control and stimulated (crocidolite or 48/80) values was significantly different (*p* < 0.05) (asterisks). CTRL = control unstimulated cells; Cro = crocidolite-stimulated cells; Wolla = wollastonite-stimulated cells; TiO_2_ = TiO_2_NW-stimulated cells; 48/80 = 48/80-stimulated cells.

**Figure 7:**
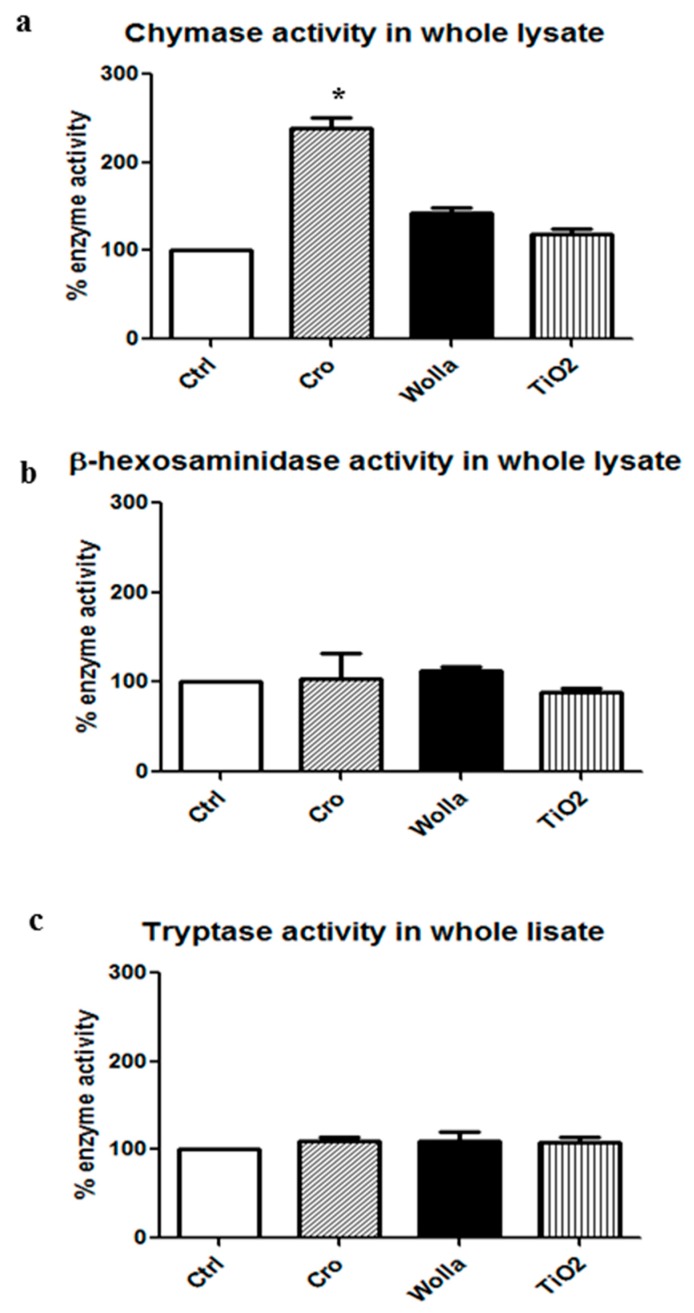
Effects of mineral fibers on enzyme activities in RPMC lysate. RPMC total lysate was obtained by sonication (3 × 10^6^ RPMC in 1 mL) and incubated for 30 min with mineral fibers (100 μg/mL). The values reported are the mean ± SD of at least five different experiments. The extent of enzyme activity was calculated taking the total enzyme activity present in the whole lysate as 100% (0.390 ± 0.052 OD/30 min for chymase; 0.160 ± 0.080 OD/30 min for β-hexosaminidase; 0120 ± 0.05 OD/30 min for tryptase (TRY)). Significant differences (*p* < 0.05) between unstimulated lysate and lysate from RPMC exposed to mineral fibers are indicated by asterisks. CTRL = unstimulated cells; Cro = crocidolite stimulated cells; Wolla = wollastonite-stimulated cells; TiO_2_ = TiO_2_NW-stimulated cells; 48/80 = 48/80-stimulated cells.

Figure 6 shows that a potent secretion was elicited by 48/80. A significant histamine release was found in the supernatant of RPMC challenged with CRO, but at a lower extent compared to 48/80. The other types of fibers elicited a weaker, but significant effect. However, despite the morphological appearance, the amount of soluble granule enzyme activity following incubation with fibers was not increased compared to that separated from unstimulated control (CTRL) cells. In the case of CHY and TRY, enzymatic activity was significantly lower (*p* < 0.05) while β-hexo appeared to be significantly released from RPMCs stimulated by TiO_2_NWs and WOLLA, but not by CRO (Table 1). This discrepancy may be explained by two possibilities: (1) an inhibitory effect exerted by the fibers on the enzymes; and/or (2) binding between fibers and either enzymes or intact granules. We therefore investigated whether asbestos fibers and TiO_2_NWs can bind and/or affect the activity of these enzymes. Figure 7a,b show that mineral fibers cannot inhibit RPMC enzymes. Surprisingly, the lysate of mast cell exhibits a significant increase in CHY activity following an incubation of 30 min with CRO. The incubation with WOLLA and TiO_2_NWs elicited a minor increment (on the average, not significant). Similarly, the activity of β-hexo and TRY was not significantly affected.

Having shown that crocidolite fibers induced significant improvement in the enzymatic activity of CHY in an RPMC lysate, we then investigated if the same effect could also be exerted on pure human enzymes. The data, summarized in Table 1, show that CRO, but also TiO_2_NWs and WOLLA, consistently increased the activity of pure human CHY and TRY, suggesting that the enhancing effect is not specific for the lysate-containing rat enzymes.

To assess if the increasing effect exerted by mineral fibers can be ascribed to the isoelectric point (IP) of the protein molecule (about 6.0–6.3 for β-hexo and TRY and 9.6 for chymase), which could influence the fiber-associating trend, we assayed the enzymatic activity of human myeloperoxidase (MPO IP, 9.3) and human eosinophil peroxidase (EPO IP, 10.8) after 30 min incubation with mineral fibers. We found that MPO activity was only weakly increased (see Supplementary Figure S8a), reaching its maximum when incubated with TiO_2_NWs, while that of EPO was significantly enhanced (Supplementary Figure S8b) specifically after incubation with crocidolite and TiO_2_NWs. These results suggest that the IP of the considered enzymes is not directly related to the increase of activity induced by fibers.

These results prompted us to evaluate the enzyme-binding capacity of mineral fibers directly by incubating them with either whole RPMC lysate or purified enzymes and monitoring the enzyme activities in the 250× *g* pellet.

**Figure 8:**
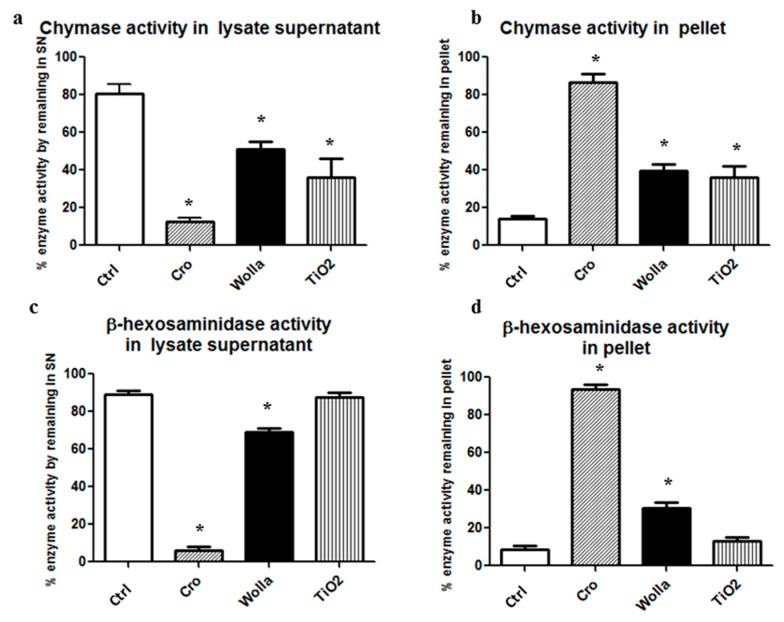
Extent of RPMC lysate enzyme absorption on mineral fibers. After 30 min incubation of the lysate in the presence of fibers (100 μg/mL), the supernatant was obtained by centrifuging the mixture at 250× *g* for 15 min at 4 °C. (**a**,**b**) fractionation of chymase activity; (**c**,**d**) fractionation of β-hexosaminidase activity. Significant changes in activity in the samples exposed to mineral fibers vs. baseline value (see the legend to Figure 7) are indicated by asterisks (*p* < 0.05). CTRL = control unstimulated cells; Cro = crocidolite-exposed enzyme; Wolla = wollastonite-exposed enzyme; TiO_2_ = TiO_2_NW-exposed enzyme.

Figure 8a–d show that the main enzyme activities in the unstimulated lysate were soluble and only a small amount was found in the 250× *g* pellet fraction, presumably due to the fact that some granules or cell aggregates escaped complete disruption. In contrast, the activity of both β-hexo and CHY were mainly found in CRO-containing pellets (86.5% and 93.6% respectively), indicating that these fibers are able to absorb both enzymes. However, the other two types of fibers also bind rat CHY and β-hexo. As much as 39.7% and 36.2% of the CHY activity was found in the WOLLA and TiO_2_NW pellets, respectively. While 30.4% of β-hexo activity was found in the WOLLA pellet, only a small fraction (12.9%) of this enzyme was found in the TiO_2_NW pellet. Comparable findings were obtained when assaying pure human chymase activity. In this case, all three types of mineral fibers were able to drag a consistent amount of the enzyme activity (over 50%) in the pellet fraction. All these data are summarized in Table 1.

## 4. Discussion

Herein, we describe the outcome of the interaction between mast cells and mineral fibers. For this purpose, we chose crocidolite, a type of asbestos which is thought to be the most active and whose mechanism of cell interaction is not yet completely clear, and TiO_2_ nanowires, which are considered to be a more active form of titanium oxide and whose industrial applications are increasing. Even for the latter, the mechanism of interaction with inflammatory cells is still only partially known. However, TiO_2_NPs are thought to be very promising for the development of new technologies [3] and are expected to be released in the environment in relatively large amounts due to their widespread use in photovoltaic cells, photoelectrolizers, and other applications.

Light microscopy analysis of the secretory process of RPMC was quickly activated after 5 min of incubation with crocidolite. This is comparable to the potent stimulus induced by the 48/80 compound. TiO_2_NWs also stimulate mast cell degranulation, but after a longer incubation period, while as expected WOLLA represents a weaker RPMC stimulus. Following TiO_2_/crocidolite-induced degranulation, the granules appear to adhere to the stimulating fibers.

This granule-to-fiber adhesion can be also induced by incubating isolated membrane-covered RPMC granules with crocidolite. The scanning electron microscopy appearance of stimulated MCs strongly suggests that both crocidolite fibers and TiO_2_NWs induce a significant secretory reaction, although to a different extent, thus confirming the preliminary light microscopy findings. SEM analysis also confirmed free granule adhesion to the mineral fibers (see Figure 2 and Figure 3) and suggests that this adhesion, which resists to all the procedures for preparing SEM samples is consistent.

Some CRO fibers are completely covered/surrounded by the cell membrane, while others protrude from the cell, as would be expected if they had pierced the cell. The detailed TEM analysis of RPMC–CRO interaction suggests two possible pathways for fiber–membrane interaction: one could be via the conventional mechanism of phagocytosis, entailing adhesion to the plasma membrane and subsequent engulfment, as reported for macrophages and other cells; the second, which has already been suggested by some authors, including ourselves, is the passive fiber-dependent piercing of the RPMC membrane resulting in the presence of nude fibers in the cytosol without any surrounding membrane. This penetration pathway has already been demonstrated, and is now widely accepted, to be the main one for TiO_2_NWs and NPs [4,6,15]. The interaction between asbestos fibers and cell membranes has been under investigation since the 1980s [86,87,88,89,90]. In general terms, it is thought that the first interaction between macrophages and asbestos fibers occurs through a phagocytic process [36], which does not exclude the possibility that the ingested fibers may exit from the phagosome into the cytosol and even enter the nuclear compartment [51,91,92], nor that passive entry of fibers through the cell membrane may occur at the same time [31,91]. Despite all this evidence, the mechanism of interaction of asbestos fibers with cell membrane phospholipids and how this influences cell membrane and intracellular organelle properties is still a matter of debate. Our findings show, for the first time, that in addition to being entrapped by pseudopodia (as in a conventional phagocytic process), fibers can also access the cell cytosol in another way. Even if the relative mechanism remains unknown, we suggest that they can passively pierce the MC membrane. While the former type of interaction could be specific and trigger a conventional secretory process, the latter could be lethal for cells, eventually leading to their disruption and to the passive release of all their granules. Direct evidence supporting a piercing action of the fiber is presently missing, but monitoring of the CRO fiber inside the cell for 120–720 nm shows that the fiber is without doubt inside the cell without any surrounding membranes (Supplementary Figures S5–S7). Further research must be carried out to investigate the mechanism that allows cytosolic subcellular localization of mineral fibers.

The ultrastructural analysis of the RPMC–CRO interaction shows that the secretory process can be carried out via two possible pathways: active secretion with single granule secretion, and conventional compound exocytosis, both of which cause progressive release of granule content and formation of granule remnants, and passive expulsion of membrane-covered granules due to plasmamembrane rupture. Our findings suggest that both these granule structures can adhere to mineral fibers, but very likely it is the latter that mainly adhere. This finding also suggests that the main pathway of “secretion” is realized via granule expulsion rather than active secretion.

The finding that the analysis by both SEM and TEM shows the extent of granule extrusion/secretion to be lower than that observed by light microscopy is probably due to the more conservative procedures employed in the processing for electron microscope analysis compared to the more disruptive procedures employed for preparation of cytocentrifuge specimens. We suspect that RPMCs may become more susceptible to disruption as a result of mechanical stress (cytocentrifugation) following exposure to mineral fibers.

As expected, histamine release was in agreement with the morphological appearance of fiber-challenged RPMCs. Conversely, despite this, the extracellular release of granular enzymes in CRO-stimulated RPMCs was not higher from that found in unstimulated control (CTRL) cells (and in the case of CHY and TRY was even significantly lower). Apparently, TiO_2_NWs and WOLLA acted as a stimulus only for β-hexo release. In the case of WOLLA, this finding is in contrast with the morphological analysis, according to which only a very weak secretion, if any, is induced by this mineral fiber. This discrepancy could be explained by the different subcellular localization of the secretory granule components (CHY) and secretory lysosome enzymes (β-hexo) [93]. The two compartments could follow different pathways of secretion, with the latter being mobilized specifically by WOLLA. On the basis of morphological analysis, we think it likely that CRO and TiO_2_NWs are, conversely, able to mobilize all secretory compartments, including granules and lysosomes. These findings were not investigated further as this would have required a series of dedicated experiments.

We excluded that the discrepancy between morphological and enzymatic analysis of the degranulation process could be ascribed to an inhibitory effect exerted by mineral fibers on soluble enzymes. On the contrary, we found that CRO significantly enhanced RPMC CHY activity, while the two other types of mineral fiber had a weaker (non-significant) activity-enhancing effect on this enzyme in RPMC lysates. Similar to the effect observed in the rat enzyme, human pure chymase activity was also significantly increased by all three fiber types. Of note, human tryptase activity was also increased by incubation with CRO. We believe it is likely that enzymatic activity enhancement may require enzyme–fiber absorption as a necessary, albeit not sufficient, step. The high isoelectric point of CHY and EPO (whose activities are greatly enhanced) vs. low IP value of β-hexo (whose activity shows weak or no enhancement), would lend support to this hypothesis. However, an electrostatic interaction cannot be the only feature involved. Firstly, the activity of MPO, which has a high IP (IP 9.3), appears to be only marginally increased; secondly, chymase is released together with heparin [94], so that the electrostatic charge of the complex is not that of the pure protein. It would be of interest to investigate the effects of the presence of heparin in the interaction between CRO and chymase. Other factors besides IP, such as molecular weight of the interacting protein and/or fiber zeta-potential, could be involved. As far as tryptase is concerned, we found that while the rat enzyme is only very weakly affected by incubation with the fibers, the human protein is significantly enhanced when incubated with crocidolite. This finding also supports a role of human mast cell protease in the pathological events induced by mineral fiber inhalation.

Taken together, these results indicate that, in the case of CRO and TiO_2_, the low activity of the granular enzyme found extracellularly, as opposed to the consistent degranulation shown by the morphological analysis and histamine release, could depend on a significant adsorption of the soluble released enzyme on the fiber and, secondly, on the binding of the granules to the fibers, which drags down the granule structures with all or part of their content. The finding that histamine, which is actually free in solution, is not bound by all the types of fibers under our experimental conditions (not shown) supports this possibility.

WOLLA, a weak stimulus for degranulation, is also able to bind soluble enzymes, thereby probably sequestering small amounts of them even from the unstimulated RPMC supernatant, but fails to bind whole granules, as shown by ultrastructural analysis. These findings suggest that different mechanisms underlie the fiber-binding capacity for soluble enzymes and membrane-covered granules.

We present herein evidence that asbestos fibers, and to a lesser extent TiO_2_NWs, are potent stimuli for MC granule secretion. Morphological evidence suggests that the fiber–cell interaction can induce cell membrane stimulus/injury, followed by the secretion of granule content or of whole granules. In either case, granular structures and/or soluble enzymes can bind to fibers and coat them. It must be noted that, as mineral fibers can sequester both granules and enzymes (or enzymes only in the case of WOLLA) at the same time, it is very difficult to quantitate precisely the amount of enzyme secreted using conventional criteria.

The improvement of the properties of immobilized protein is a well-known process. Enzymes loaded on a carrier display prolonged half-life, improved stability, and acquire a range of specific advantages, such as broad optimal pH and temperature range [95]. A similar improvement of the properties of immobilized chymase could explain the higher enzymatic activity of this protein observed in our experiments. We think it is possible that the granule content is continuously solubilized from the granule and recaptured /bound again by the fiber. Chymase, which displayed both an activity-enhancing effect and high fiber-binding capacity, may significantly increase the pro-inflammatory capacity of mineral fibers (particularly of asbestos). More specifically, chymase can activate IL-1β and potentiate the effect of histamine [96], as well as degrade IL-33, giving rise to mature forms with a potent effect on innate lymphoid cells [97], and eventually degrading it completely [98]. The activity of these IL-1 family members should improve when the peptidase activity of mast cells bound to mineral (particularly asbestos) fibers is stimulated. We also expect the activity of immobilized enzymes to become more stable, which would contribute to their prolonged proinflammatory capacity. Notably, TiO_2_, which has already been used to immobilize enzymes, and wollastonite are able to bind a variety of enzymes and improve their activity. However, their capacity in this sense is significantly lower than that of crocidolite. Since asbestos fibers persist in human lung and pleura [99] together with MC [65] for decades, the granule/enzyme-enriched fibers induced by both CRO and TiO_2_, could prolong their activity over long periods of time. Data on the toxicity of nanowires are still very limited. Our results suggest that certain nanowires, such as TiO_2_NWs, may present health risks similar to those posed by asbestos fibers. Therefore, it is important that the toxicity of all inorganic nanofilaments are systematically assessed before fully allowing their industrial application and mass production. 

These results highlight the critical role that MCs may play in the pathogenesis of mineral fiber-related diseases. Mast cells (either mucosal and connective tissue type) are present in human airways [67]. This cell population is equipped with histamine, tryptase, and chymase, which following mineral fiber-induced release may be involved in progression of fibrosis and the inflammatory process. Our model focusing on RPMCs, which are of the connective type (MCct), cannot directly demonstrate the involvement of human mast cells in these pathological events, which are typical of mineral fiber-induced disease. However, the findings that: (1) Th2 response is active in exposed subjects; (2) both human and rat MC protease (CHY and TRY) activities are improved by the contact with mineral fibers, particularly asbestos; and (3) in the human airway MCct are present [67], together suggest that RPMCs could be a valid surrogate of human mast cells of the connective type and that the latter may also be involved in mineral fiber-induced pathology in humans. However, further experiments in an animal model are needed to directly investigate the proinflammatory role played by protease-coated fibers vs their uncoated counterparts. In this model, we can also evaluate the long-term (months) effect of mineral fibers on cytokine expression, synthesis, and release. Such granule/enzyme-enriched fibers resemble mast cell extracellular traps (MCETs) [100], which are known to increase and maintain a pro-inflammatory stimulus in tissues. We suggest that the stimulus of Th2 immunity exerted by the crocidolite fibers and, to a lower extent by TiO_2_NPs, can be ascribed to the outcomes of the fiber–mast cell interaction described herein, and that this interaction should be considered for the identification of new therapeutic targets to treat exposed subjects.

## 5. Conclusions

In conclusion, the novel findings reported in this paper can be summarized as follows:

1. Mineral fibers are a potent stimulus for the mast cell secretory process, through both active (during membrane interaction) and passive (during cytosolic penetration) interactions. The rank order is: CRO > TiO_2_NW >> WOLLA. We demonstrate by serial sections at TEM analysis that the cytosolic inclusion of asbestos fibers is not an artifact, but that they are actually inside the cell. We therefore speculate that the presence of fibers inside the cytosol may be toxic and trigger cell rupture and granule expulsion. Of note, TiO_2_ nanoparticles were also found inside the cytosol without any surrounding membrane.
2. Tissue-deposited fibers can aggregate large amounts of pro-inflammatory factors by binding free enzymes, intact granules, and granule remnants. Deposited fibers can continue to bind granules from MCs as cells are renewed and more fibers are inhaled, thereby creating a long-lasting pro-inflammatory environment.
3. MCs could contribute to the inflammatory and toxic effects associated with some engineered nanomaterials, including TiO_2_NPs, which are widely used and are therefore a cause of increasing concern for their potential to harm humans.

## Figures and Tables

**Table 1:** Summary of fiber–enzyme interactions.

Isoelectric Point		Chymase Activity				β-Hexosaminidase Activity				Tryptase Activity			
	(IP)	9.6				5.6–6.1				6.0–6.3			
		CTRL	CRO	WOLLA	TiO_2_	CTRL	CRO	WOLLA	TiO_2_	CTRL	CRO	WOLLA	TiO_2_
RPMC	% Release	16.5 ± 3.8	9.4 ± 2.6 *	8.2 ± 3.2 *	12.00 ± 2.6	14.2 ± 5.2	8.1 ± 5.1	41.0 ± 3.6 *	39.1 ± 2.9 *	19 ± 3.0	13.0 ± 1.5	8.0 ± 2.0	10.5 ± 1.8
Lysate	% increment	100	238.5 ± 19.6 *	143.0 ± 7.1	125.0 ± 7.1	100	103.3 ± 51.3	112.0 ± 7.0	88.00 ± 10.2	100	125 ± 6.0	110 ± 12.2	105 ± 5.0
	% pellet activity	13.8 ± 2.5	86.5 ± 7.7 *	39.7 ± 6.1 *	36.2 ± 10.1 *	8.6 ± 3.5	93.6 ± 4.1 *	30.4 ± 5.5 *	12.9 ± 4.0	nd	nd	nd	nd
Human pure enzymes	% increment	100	224.9 ± 44.6 *	244.6 ± 50.8 *	244.7 ± 8.3 *	nd	nd	nd	nd	100	160 ± 10.0	97.0 ± 12.0	110.0 ± 6.0
	% pellet activity	8.1 ± 2.1	68.9 ± 11.7 *	61.8 ± 1.9 *	54.3 ± 9.1 *	nd	nd	nd	nd	nd	nd	nd	nd

% Release: refers to the amount of enzyme released from RPMCs after mineral fiber exposure found in the 250× *g* supernatant, taking the total amount of the RPMC enzyme as 100%; % increment: refers to the percentage of increased enzyme activity in the presence of fibers taking the activity of the untreated enzyme as 100%. The differences between resting lysate and lysate from RPMCs exposed to mineral fibers were statistically significant when indicated by asterisks (*p* < 0.05). IP: isoelectric point; nd: not determined.

## Supplementary Materials

The following are available online at www.mdpi.com/1660-4601/15/1/104/s1, Figure S1: Crocidolite characterization by ultrastructural and EDX analysis; Figure S2: Titanium dioxide nanowire characterization by ultrastructural and EDX analysis; Figure S3: Wollastonite characterization by ultrastructural and EDX analysis; Figure S4: Scanning electron microscope appearance of membrane-covered granules and granule remnants; Figure S5: Serial sections of the same RPMC spanning 120 nm in depth; Figure S6: Serial sections of the same RPMC spanning 720 nm in depth; Figure S7: Serial sections of the same rat peritoneal eosinophil spanning 300 nm in depth; Figure S8: Effects of the interaction of mineral fibers with pure human peroxidases.

## Abbreviations

BSA	bovine serum albumin
MCs	mast cells
RPMCs	rat peritoneal mast cells
β-hexo	β-hexosaminidase
CHY	chymase
TRY	tryptase
CRO	crocidolite
TiO_2_	titanium oxide
NWs	nanowires
NPs	nanoparticles
WOLLA	wollastonite fibers
NLRP3	NLR pyrin domain-containing 3
PBS	phosphate buffered saline
SEM	scanning electron microscope
TEM	transmission electron microscope
IP	isoelectric point
hMPO	human myeloperoxidase
hEPO	eosinophil peroxidase
MCETs	mast cell extracellular traps
TMB	3,3′,5,5′-Tetramethylbenzidine
TB	trypan blue
SN	supernatant
P	pellet
ROS	reactive oxygen species
