# Supplementary material for: The Secretory Response of Rat Peritoneal Mast Cells on Exposure to Mineral Fibers
Source: Int J Environ Res Public Health. 2018 Jan 10;15(1):104. doi: 10.3390/ijerph15010104 (PMC5800203; doi:10.3390/ijerph15010104)
Supplement: Supplementary file 1 [file ijerph-15-00104-s001.pdf]

International Journal of
Environmental Research and Public Health

MDPI

Supplementary Material

The Secretory Response of Rat Peritoneal Mast Cells on Exposure to Mineral Fibers

Violetta Borelli, Elisa Trevisan, Vita Francesca and Giuliano Zabucchi

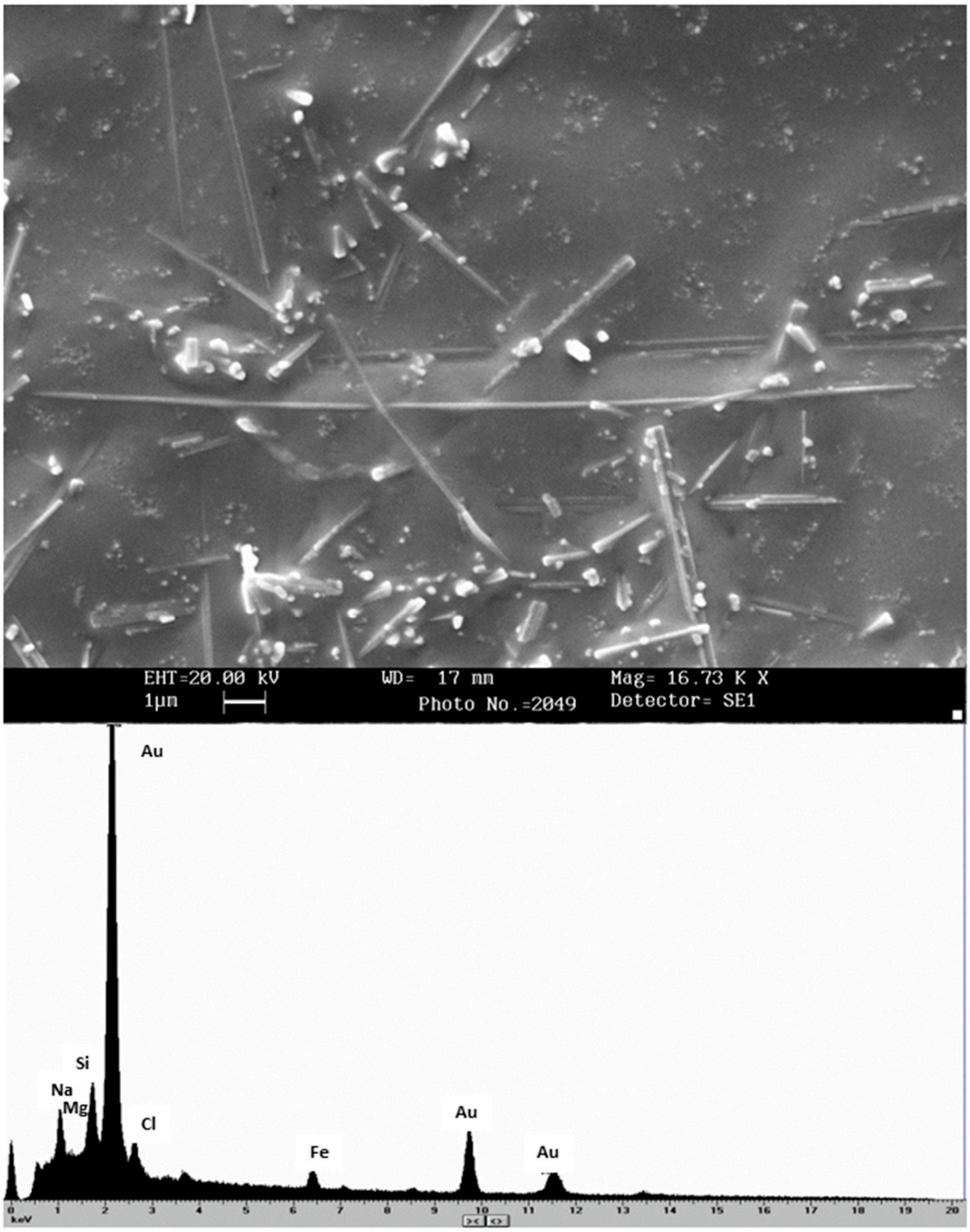

Figure S1. **Crocidolite characterization by ultrastructural and EDX analysis.** The ultrastructural morphology (upper panel) and chemical composition (lower panel) of the mineral fibers is shown. The chemical composition of crocidolite was found compatible with that already reported by other authors (see Material and Method section). The detailed morphological analysis carried out on at least one hundred fibers showed that crocidolite fibers has on the average a length of 9.2 ± 1.5 (SE) (range min–max 2.1–35.7) µm.

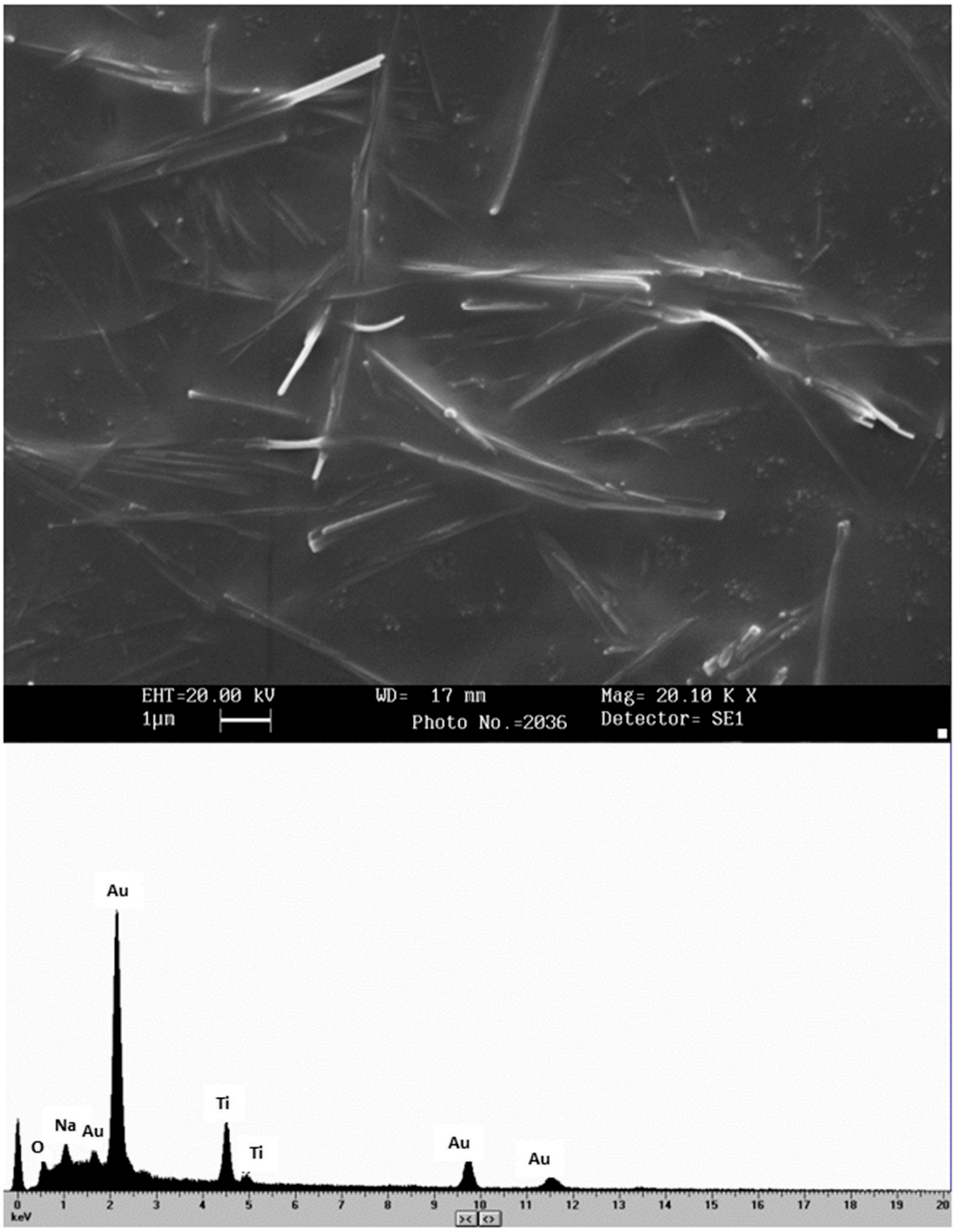

Figure S2. **Titanium dioxide nanowires characterization by ultrastructural and EDX analysis. The ultrastructural morphology** (upper panel) and chemical composition (lower panel) of the mineral fibers is shown. Nanowires were prepared from a mixture of two mineral forms of TiO_2, rutile and anatase. The chemical composition TiO_2NW was found compatible with that already reported by other authors (see Material and Method section). The detailed morphological analysis carried out on at least one hundred fibers showed that TiO_2 NW fibers, has on the average, a length of 2.9 ± 0.5 (SE) (range min–max 1.0-20.2) µm.

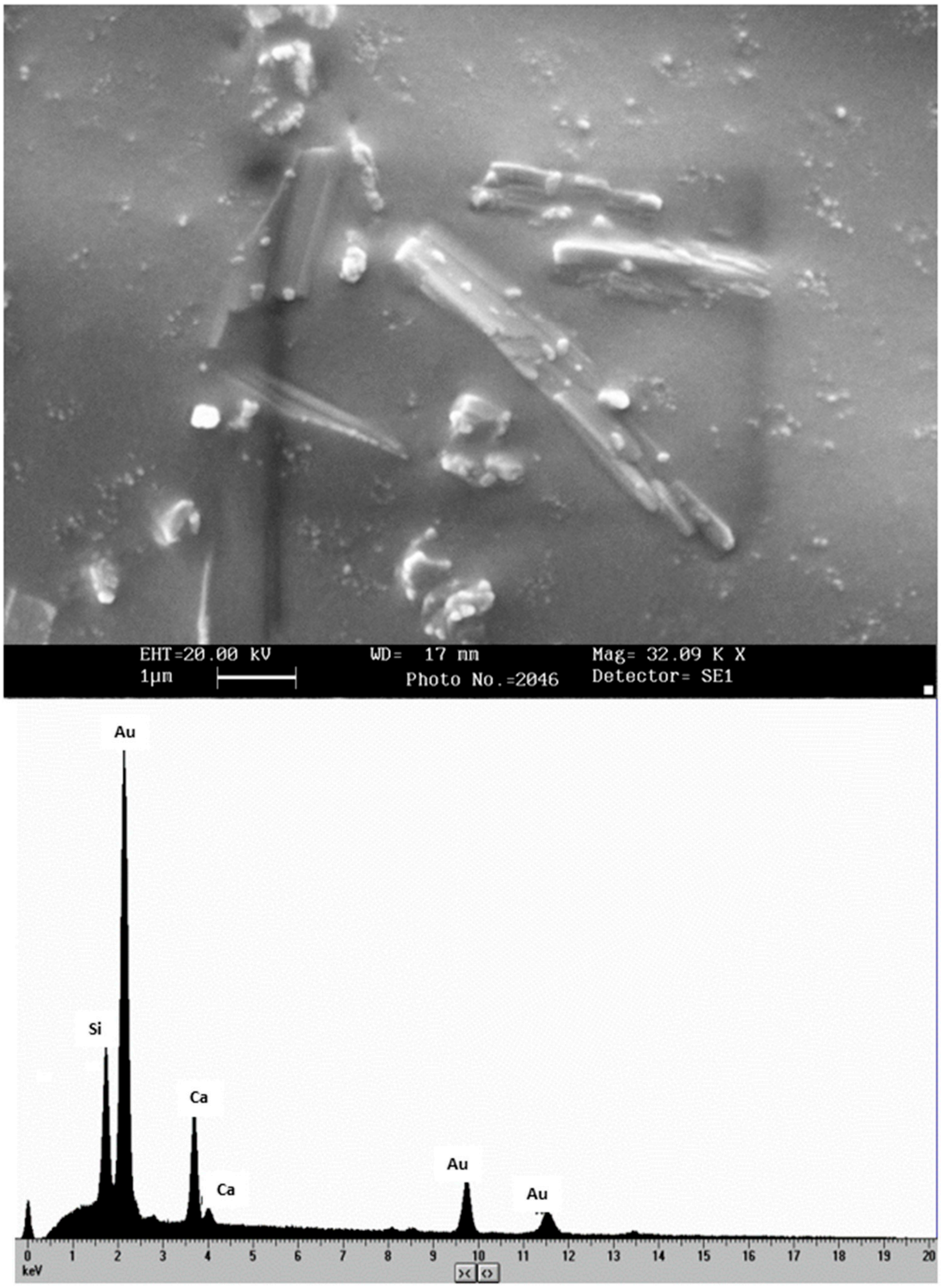

Figure S3. Wollastonite characterization by ultrastructural and EDX analysis. The ultrastructural morphology (upper panel) and chemical composition (lower panel) of the mineral fibers is shown. As expected WOLLA (a Calcium inosilicate) was revealed to be mainly composed of calcium and silicium**.** The detailed morphological analysis carried out on at least one hundred fibers showed that wollastonite fibers has on the average a length of 4.2 ± 0.79 (SE) (range min–max 1.7–27.6) µm.

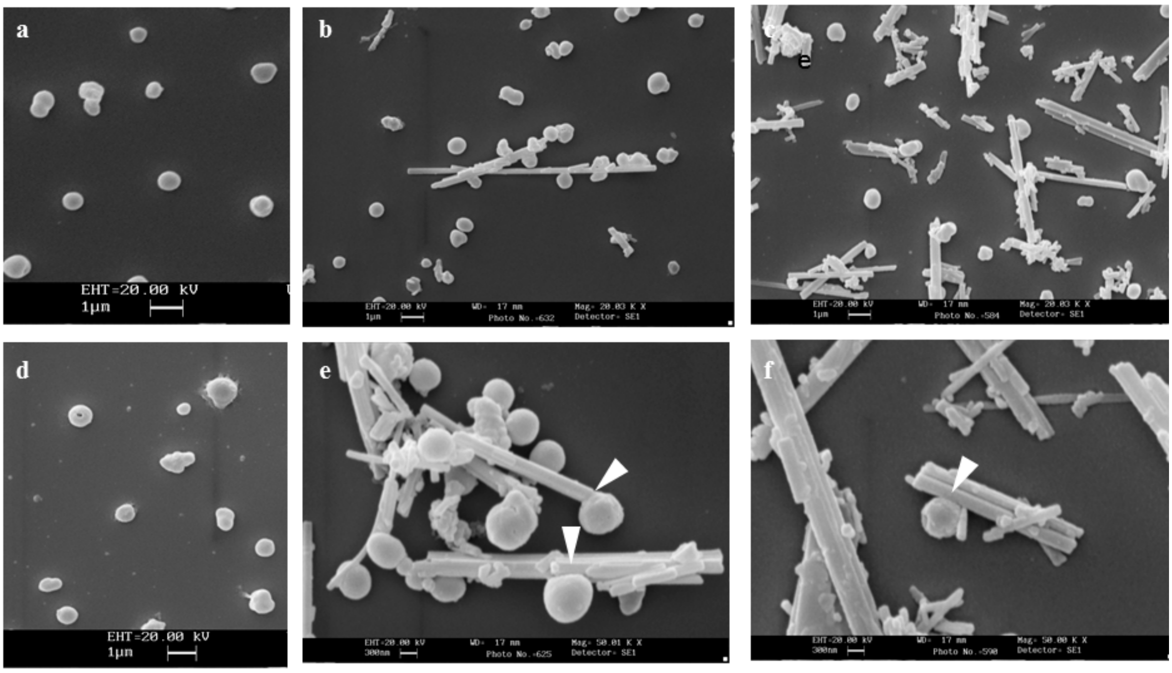

Figure S4. Ultrastructural (SEM) appearance of membrane- covered isolated granules and isolated granule remnants. Membrane covered granules, isolated with mild sonication (see Materials and Methods) are shown in panel a. Granule remnants, isolated by mild 48/80 stimulation (see Materials and Methods) are shown in panel d. Granules were immediately fixed after isolation and processed for ultrastructural analysis. The granule remnants appear similar to the membrane covered counterpart, however a detailed analysis showed that they were larger (0.80 ± 0.02 SE m vs 0.70 ± 0.02 SE m $p < 0.005$). In panel b and c the adhesion of membrane covered granules (b) and granule remnants (c) to crocidolite fibers is shown respectively. On the average the affinity of membrane covered granules is about 10 fold higher with respect to that shows by granule remnants. Higher magnification analysis showed that membrane covered granules adhere intimately with the fiber (arrowheads in panel e), while granule remnants maintain their round morphology (arrowhead in panel f).

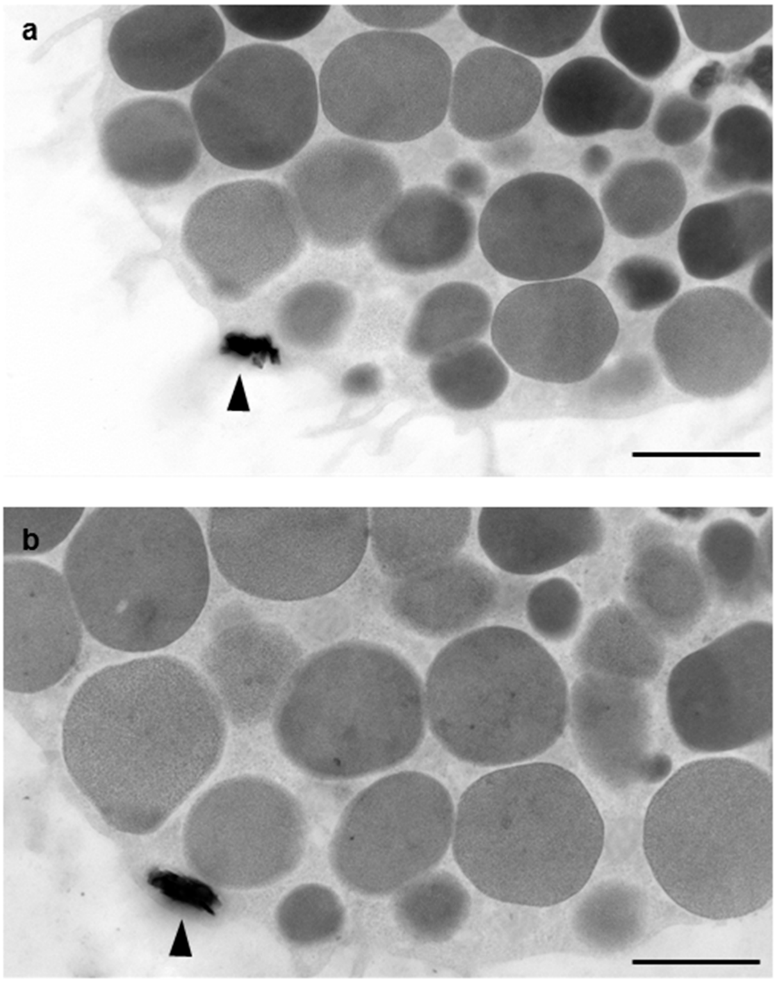

Figure S5. Serial sections of the same RPMC spanning 120 nm in depth. Black arrowheads show that in each picture one cytosol free asbestos fiber is shown in the same site of the cell. a) starting zero level; b) 120 nm deep. Magnifications: bars = 1 μm.

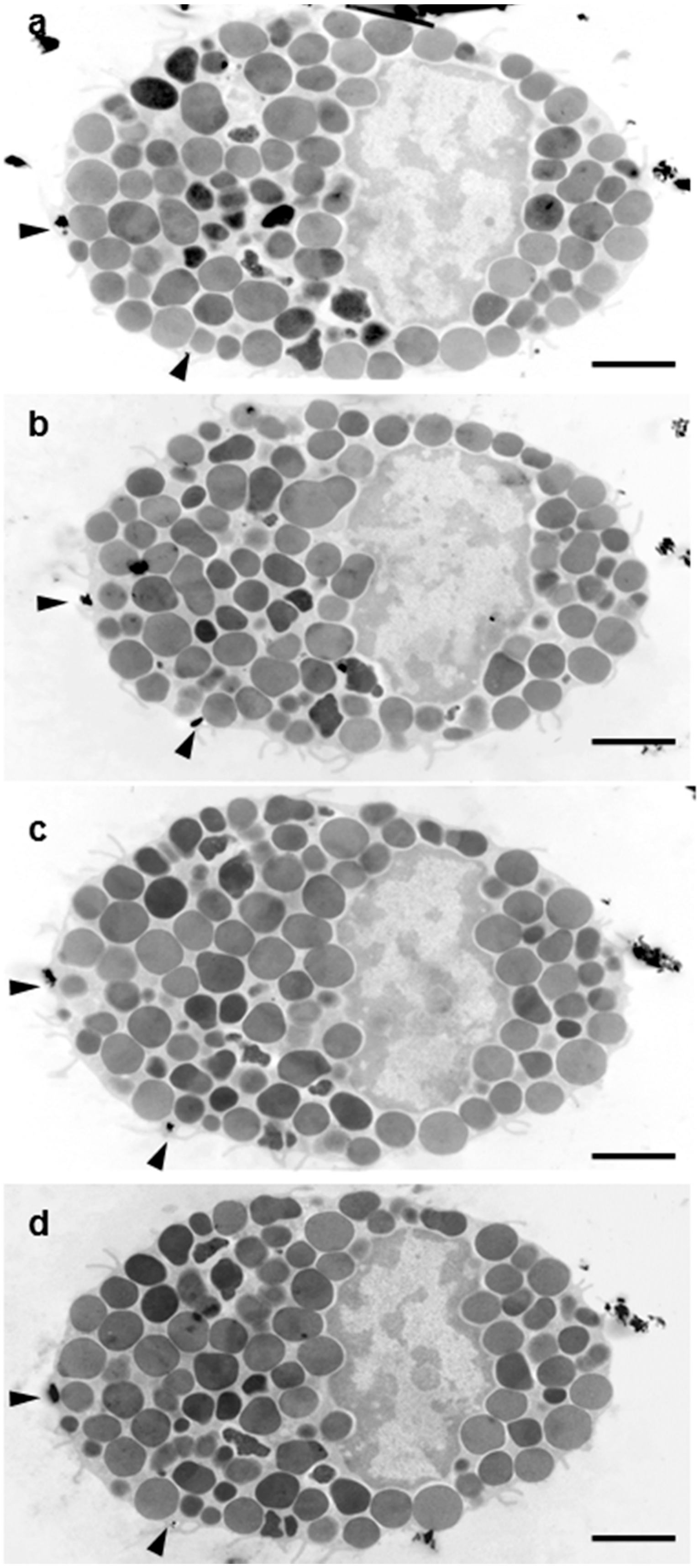

Figure S6. Serial sections of the same RPMC spanning 720 nm in depth. Black arrowheads show that in each picture two cytosol free asbestos fibers are shown in the same cell site. a) starting zero level; b) 360 nm deep; c) 600 nm deep; d) 720 nm deep. Magnifications: bars = 2 µm.

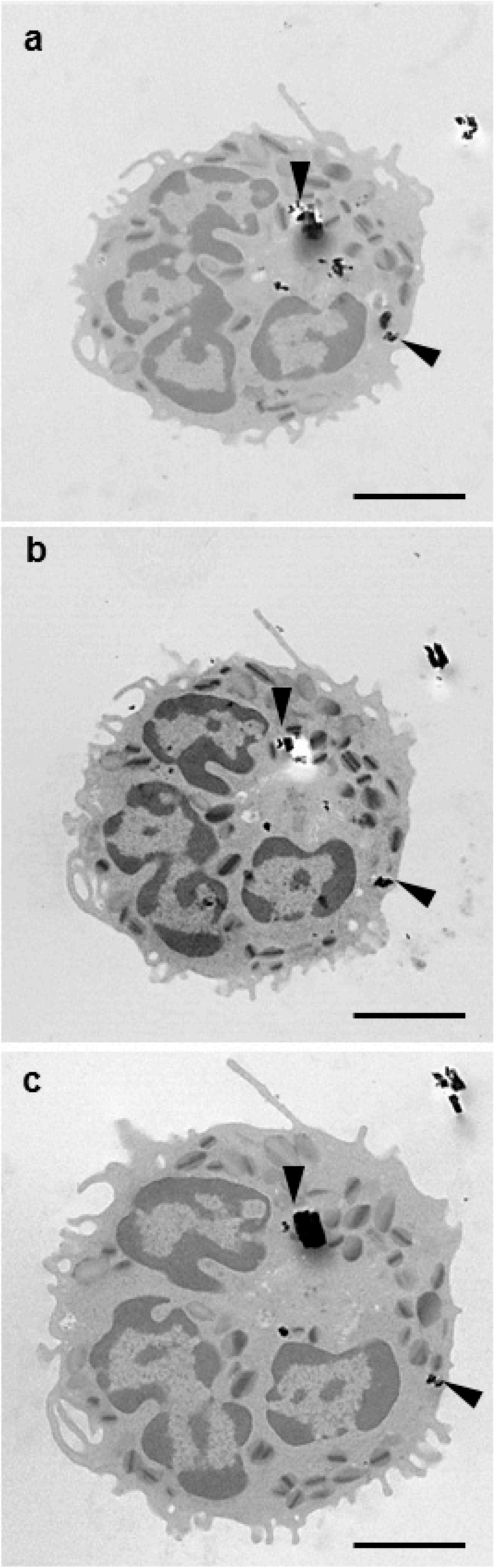

Figure S7. Serial sections of the same eosinophil spanning 300 nm in depth. Black arrowheads show that in each picture two cytosol free asbestos fiber in the same site of the cell. a) starting zero level; b) 150 nm; c) 300nm. Magnifications: bars = 2 μm.

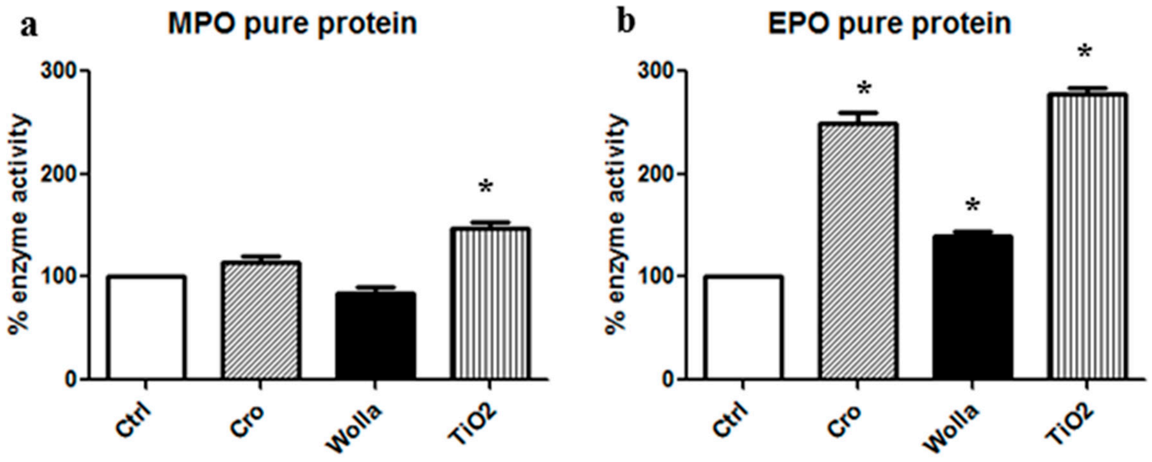

Figure S8. Effects of the interaction of mineral fibers with pure human enzymes. Human enzymes were incubated for 30 min with mineral fibers (100 μg/ml). The supernatant was obtained by centrifuging the mixture at 250 xg for 15 min at 4°C. a) myeloperoxidasae (MPO) and b) eosinophilperoxidase (EPO). The values reported are the mean ± SD of at least five different experiments. The extent of enzyme activity was calculated taking the total enzyme activity present in the no treated samples as 100% (0,116±0,020 for MPO and 0.200 for EPO; OD/2min of reaction). The statistically significant ($p<0.05$) changes in activity in the samples exposed to mineral fibers vs the baseline value, are indicated by asterisks. Ctrl= starting enzyme activity; CRO= crocidolite exposed enzyme; WOLLA= wollastonite exposed enzyme; TiO_2 = TiO_2NW exposed enzyme.
